# Effectiveness of monthly and bimonthly follow-up of patients with well-controlled type 2 diabetes: a propensity score matched cohort study

**DOI:** 10.1186/s12902-019-0372-5

**Published:** 2019-05-02

**Authors:** Tomohiko Ukai, Shuhei Ichikawa, Miho Sekimoto, Satoru Shikata, Yousuke Takemura

**Affiliations:** 1 Department of Community Medicine, TSU, Mie University School of Medicine, 2-174 Edobashi, Tsu, Mie 514-8507 Japan; 2 Division of Public Health, Osaka Institute of Public Health, 1-3-69 Nakamichi, Higashinari, Osaka, 537-0025 Japan; 3 Department of Education and Research in Family and Community Medicine, Mie University Graduate School of Medicine, 2-174 Edobashi, Tsu, Mie 514-8507 Japan; 4 Research Center for Health Policy and Economics, Hitotsubashi Institute for Advanced Study, 2-1-2 Hitotsubashi, Chiyodaku, Tokyo, 101-8439 Japan; 5 Department of Family Medicine, Mie Prefectural Ichishi Hospital, 616 Minamiieki, Hakunsan-cho, Tsu, Mie 515-3133 Japan; 6 Department of Family Medicine, MIE, Mie University School of Medicine & Graduate School of Medicine, 2-174 Edobashi, Tsu, Mie 514-8507 Japan

**Keywords:** Diabetes mellitus, Follow-up interval, Propensity score

## Abstract

**Background:**

On average, patients in Japan with type 2 diabetes mellitus have a clinical consultation every month, although evidence for a favorable follow-up interval is lacking. This study investigated whether the follow-up interval can be extended by comparing the clinical outcomes and cost for monthly versus bimonthly follow-up of patients with well-controlled diabetes mellitus.

**Methods:**

We combined administrative claims data from the National Health Insurance and the Health Checkups Program data of Tsu city, Japan between 2011 and 2014 to conduct a retrospective cohort study of patients with well-controlled type 2 diabetes mellitus. Propensity scores were used to assemble a matched-pairs cohort from patients who had monthly and bimonthly follow-up. Equivalence between two groups was assessed by designating the proportion of patients who maintained good control of their diabetes in the subsequent year as a primary outcome. The proportion achieving target blood pressure and lipid levels, favorable lifestyle, and annual cost were compared as secondary outcomes.

**Results:**

Of 12,145 participants, 693 with monthly follow-up and 693 with bimonthly follow-up were matched using propensity scores. In the monthly follow-up group 654 (94.4%) remained under good diabetic control, versus 658 (95.0%) in the bimonthly group (difference: 0.6%; 95% confidence interval: − 1.8 to 2.9%). All secondary outcomes were equivalent for the monthly and bimonthly follow-up groups except the proportion achieving target blood pressure, the proportion engaging in regular exercise, and annual cost.

**Conclusions:**

For patients with well-controlled diabetes mellitus, although frequent follow-up by a physician does not affect the control of blood glucose level in the subsequent year, the annual treatment cost becomes much higher. We suggest that patients with well-controlled diabetes can be followed up less often.

## Background

The number of physician consultations in Japan is considerable. According to the Organization for Economic Co-operation and Development (OECD) health statistics, on average Japanese individuals have a clinical consultation 12.9 times in a single year versus the OECD average of 6.6 in 2013 [1]. With regard to diabetic care, patients on average have a consultation every 33.7 days whereas guidelines across many countries recommend that patients be followed up every 3 months [2–4]. These figures indicate from a positive aspect that Japan has excellent access to health care services, but from a negative viewpoint that health services are overused.

Frequent follow-up is an attribute of Japan’s health care system. Japan has adopted a universal health insurance coverage system whereby payment for health services is decided on and controlled by the government [5]. For outpatient services, payment is made under a fee-for-service system. In addition to the payment for individual services, the government allows physicians to receive a monthly lifestyle-associated disease management fee as long as they conduct a consultation with a patient at least once a month. This system offers a financial incentive to physicians for frequent follow-up.

Frequent follow-up can be justified if it produces positive patient outcomes, but evidence concerning follow-up intervals for patients with diabetes mellitus is limited and conflicting [6]. Of two cross-sectional studies, one demonstrated an association between follow-up interval and meeting treatment goals [7] while the other showed that the number of follow-ups was not related to glycemic control [8]. In one randomized controlled trial (RCT), the monthly follow-up group had significantly better outcomes than the 3-monthly follow-up group regarding patients’ quality of life and clinical indicator values [9]. Another RCT comparing 3-monthly with 6-monthly follow-up by nurse practitioners for patients with controlled diabetes mellitus did not show an equivalent result, but the difference was not of clinical significance [10]. To date no consensus has been reached regarding the optimal follow-up interval for diabetes.

In this study we aimed to investigate whether Japan’s common clinical practice of monthly follow-up for patients with type 2 diabetes mellitus can be replaced by longer follow-up intervals. Our clinical question was as follows: is a bimonthly visit equivalent to a monthly visit regarding clinical outcomes and costs for patients with type 2 diabetes? To answer the question, we conducted a propensity score matched retrospective cohort study using the claims database of National Health Insurance and the Specified Health Checkups database.

## Methods

This study was approved by Research Ethical Committee of Mie University School of Medicine (Number 1475).

### Data sources and study population

Datasets were constructed from the claims database of National Health Insurance and The Specific Health Checkups database, both owned by Tsu city, which has 285,000 residents with 27.7% of people aged 65 years or more [11], which represents the Japanese average demographic. An opt-out sampling, in which participants’ anonymized data are used but they are free to ask for exclusion of their information from the analysis, was applied. The National Health Insurance comprises approximately 40% of the total population and is mostly intended for the self-employed, retired, and employees who are not provided the health insurance by the company [12]. The claims database of the National Health Insurance contains detailed information such as diagnoses, age, sex, data on consultation for outpatient services, procedures, and drugs provided, with information regarding dates and volume [13]. The Specific Health Checkups program is provided by the National Health Insurance annually to persons older than 40 years and focuses on detecting risk factors for lifestyle diseases [14]. The program consists of regular blood and urine tests, standardized interviews by public health nurses, and physical examinations by physicians.

Patients with a diagnosis of type 2 diabetes mellitus who made use of the Specific Health Checkups program between 2011 and 2014 were studied. We included patients with good hemoglobin A1c control (HbA1c ≤ 7.0%) in the Specific Health Checkup. We excluded non-well-controlled diabetes patients because they need frequent follow-up and hence potentially engender reverse causality. We also excluded patients receiving intensive insulin treatment and those with type 1 diabetes mellitus because current guidelines recommend that these patients should have frequent monitoring regardless of diabetic control [15].

We set the follow-up interval as exposure. The follow-up intervals for diabetic care were extracted from the claims database of National Health Insurance. Since the average follow-up interval is 33.7 days and the largest population of type 2 diabetes patients has a monthly consultation followed by bimonthly consultation [2], patients with monthly and bimonthly follow-ups were compared.

The primary outcome was the proportion of patients who maintain good HbA1c control defined as HbA1c ≤ 7.0% in the results of the Specific Health Checkups in the subsequent year. The secondary outcomes included the percentage of persons who maintained good blood pressure control, defined as systolic blood pressure ≤ 140 mmHg and diastolic blood pressure ≤ 90 mmHg; who maintained good cholesterol control, defined as low-density lipoprotein (LDL) cholesterol ≤140 mg/dL, high-density lipoprotein (HDL) cholesterol ≥34 mg/dL, and triglyceride ≤300 mg/dL; and whose HbA1c, blood pressure, and cholesterol were all well controlled. Other secondary outcomes were HbA1c, systolic blood pressure, diastolic blood pressure, LDL cholesterol, HDL cholesterol, and triglyceride on a continuous scale; body mass index (BMI); the percentage of current smokers; the percentage of those undertaking regular exercise; the percentage of those with daily alcohol intake; and those who sleep well. The total amounts of money claimed annually for diabetic care were also compared.

From the Specific Health Checkups database, patients’ background data including age, sex, BMI, smoking habit, regular exercise, drinking habit, sleeping status, past medical history, medication, blood pressure, and blood test results (HbA1c, LDL cholesterol, HDL cholesterol, triglyceride, aspartate aminotransferase (AST), alanine aminotransferase (ALT), γ-glutamyl transpeptidase (GGT), uric acid, albumin, estimated glomerular filtration rate (eGFR)) were collected. We also collected data regarding the dates of physician visits and diagnosis and the costs for each visit from the claims database of National Health Insurance.

### Statistical methods

The propensity score analysis was used to assess differences between monthly follow-up and bimonthly follow-up. A multivariable logistic regression model was developed to estimate a propensity score for follow-up interval using all clinically relevant variables. Participants in the monthly follow-up group were matched to those in the bimonthly group based on propensity scores. A 1-to-1 matching algorithm without replacement was adopted with the nearest neighbor matching within the calipers of width equal to 0.20 of the standard deviation of the logit of the propensity score. We used an iterative approach to refine the logistic regression model to achieve a balance of covariates between the matched pairs. We calculated the standardized difference to measure covariate balance, with standardized difference above 10% regarded as meaningful imbalance.

We calculated the difference in percentage of good diabetic control between the monthly and bimonthly groups, with the corresponding confidence interval (CI) using method 10 of Newcombe [16]. This CI was compared with the prespecified range of equivalence. We assumed that equivalence was ascertained if the two-sided 95% CI for the difference in categorical outcomes was completely in the range − 5 to 5% between the two groups [17]. For continuous outcomes, we calculated the effect size, i.e., the average difference between groups divided by the standard deviation. We assumed that equivalence was ascertained if the effect size was 0.3 or less [18].

## Results

**Fig. 1:**
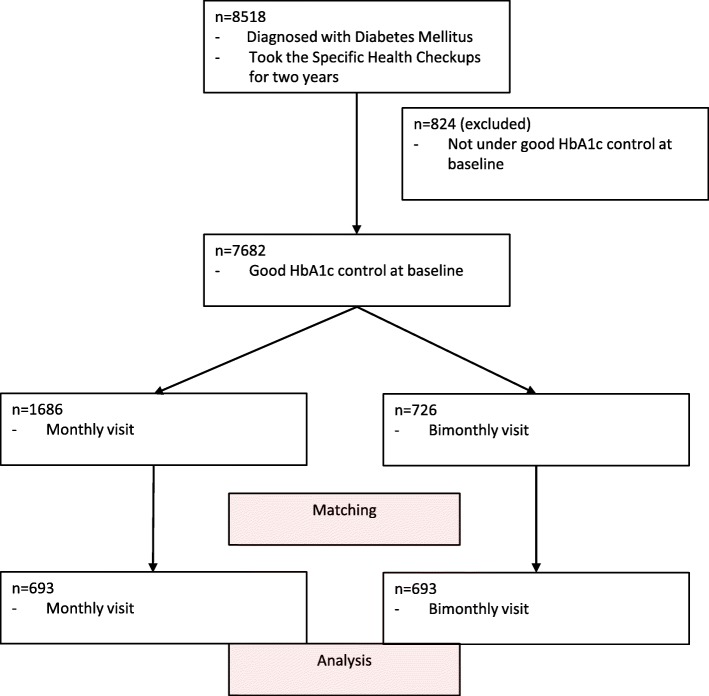
Flowchart of selecting participants for analysis

The study cohort included 12,145 samples from 8518 individuals with type 2 diabetes mellitus who undertook the Specific Health Checkups. Information about the Specific Health Checkups of the subsequent year was available for 10,124 (83.3%). The Specific Health Checkups at baseline showed 7682 patients under good HbA1c control. Among these, 1686 visited physicians monthly and 726 visited bimonthly (Fig. 1).

Before propensity score matching, there was a significant difference between the two groups (Table 1). The absolute standardized difference was greater than 10% for BMI, HbA1c, LDL cholesterol, and the proportions of antihypertensive drug users, lipid-lowering drug users, and oral blood glucose users. After propensity score matching, participants in the monthly and bimonthly follow-up groups were well balanced and the absolute standardized difference was within 10% in all covariates (Table 2). In total, 693 monthly follow-up and 693 bimonthly follow-up patients were compared.

**Table 1:** Characteristics of the study population who received monthly follow-up or bimonthly follow-up

	Monthly Visit (*n* = 1686)	Bimonthly Visit (*n* = 726)	Absolute Standardized Difference, %	*P* Value
Demographics				
Age	66.5 ± 5.0	66.3 ± 5.2	4.2	0.35
Gender (female)	812 (48.2)	352 (48.5)	0.6	0.88
BMI	24.0 ± 3.6	23.4 ± 3.2	18.0	< 0.001
Current smoker	190 (11.3)	77 (10.6)	2.1	0.63
Regular exercise	856 (51.0)	363 (50.1)	1.7	0.70
Daily alcohol intake	350 (20.8)	159 (22.0)	2.9	0.23
Sleep well	1496 (89.4)	627 (86.7)	8.3	0.06
Skip breakfast more than three times in a week	49 (2.9)	23 (3.2)	1.5	0.73
Medication				
Antihypertensive Drugs	1094 (64.9)	366 (50.4)	29.6	< 0.01
Oral Blood Glucose Lowering Drugs	1359 (80.6)	433 (59.6)	39.9	< 0.01
Lipid Lowering Drugs	845 (50.1)	291 (40.1)	20.3	< 0.01
Medical History				
History of celebrovascular diseases	87 (5.2)	42 (5.8)	2.8	0.53
History of heart diseases	161 (9.5)	89 (12.3)	8.7	0.05
History of renal failure	9 (0.5)	1 (0.1)	6.9	0.17
CKD				
stage 1	232 (13.8)	77 (10.6)	9.7	0.03
stage 2	1165 (69.1)	519 (71.5)	5.2	0.24
stage 3	280 (16.6)	127 (17.5)	2.4	0.59
stage 4	7 (0.4)	2 (0.3)	2.4	0.61
stage 5	1 (0.1)	1 (0.1)	2.5	0.54
Systlic Blood Pressure (mmHg)	130.9 ± 14.8	129.9 ± 15.9	6.7	0.13
Diastlic Blood Pressure (mmHg)	74.4 ± 9.6	74.8 ± 9.7	3.5	0.43
HbA1c (%)	6.2 ± 0.5	6.0 ± 0.5	31.3	0.00
LDL (mg/dl)	116.2 ± 27.2	119.0 ± 28.7	10.2	0.02
HDL (mg/dl)	58.5 ± 15.8	59.1 ± 14.9	3.9	0.38
TG (mg/dl)	127.7 ± 71.9	122.8 ± 69.2	7.0	0.12
GOT (IU/L)	25.0 ± 10.2	24.6 ± 9.4	3.9	0.39
GPT (IU/L)	24.1 ± 13.7	23.3 ± 14.4	5.6	0.20
G-GTP (IU/L)	38.8 ± 39.7	36.1 ± 37.2	7.1	0.11
Uric acid (mg/dl)	5.5 ± 1.4	5.5 ± 1.3	2.8	0.53
eGFR (mL/min/1.73 m2)	73.5 ± 15.3	72.2 ± 15.0	8.4	0.06
Albmin (g/dl)	4.3 ± 0.2	4.3 ± 0.3	1.5	0.74
Hemoglobin (g/dl)	13.8 ± 1.4	13.9 ± 1.4	11.4	0.01

*SD* Standard deviation, *BMI* Body mass index, *HbA1c* Hemoglobin A1c, *LDL* Low-density lipoprotein, *HDL* High-density lipoprotein, *TG* Triacylglycerol, *AST* Aspartate aminotransferase, *ALT* Alanine aminotransferase, *GGT* γ-glutamyl transpeptidase, *eGFR* Estimated glomerular filtration rate, *CKD* Chronic kidney disease

**Table 2:** Characteristics of the propensity score matched pairs

	Monthly Visit (*n* = 696)	Bimonthly Visit (n = 696)	Absolute Standardized Difference, %	P Value
Demographics				
Age	66.3 ± 5.0	66.3 ± 5.1	0.3	0.95
Gender (female)	329 (47.5)	334 (48.2)	1.4	0.79
BMI	23.4 ± 3.0	23.4 ± 3.2	0.4	0.93
Current smoker	63 (9.1)	71 (10.3)	3.7	0.47
Regular exercise	334 (48.2)	348 (50.2)	4.0	0.45
Daily alcohol intake	141 (20.4)	150 (21.7)	3.2	0.97
Sleep well	595 (85.9)	598 (86.3)	1.3	0.82
Skip breakfast more than three times in a week	20 (2.9)	21 (3.0)	0.8	0.87
Medication				
Antihypertensive Drugs	363 (52.4)	344 (49.6)	5.6	0.31
Oral Blood Glucose Lowering Drugs	430 (62.0)	414 (59.7)	5.0	0.35
Lipid Lowering Drugs	292 (42.1)	281 (40.6)	3.2	0.55
Medical History				
History of celebrovascular diseases	38 (5.5)	40 (5.8)	1.3	0.82
History of heart diseases	92 (13.3)	84 (12.1)	3.7	0.52
History of renal failure	0 (0.0)	1 (0.1)	2.5	0.32
CKD				
stage 1	64 (9.2)	70 (10.1)	2.6	0.59
stage 2	492 (71.0)	498 (71.9)	1.9	0.72
stage 3	136 (19.6)	122 (17.6)	5.4	0.33
stage 4	1 (0.1)	2 (0.3)	2.5	0.56
stage 5	0 (0.0)	1 (0.1)	4.6	0.32
Systlic Blood Pressure (mmHg)	129.6 ± 15.6	129.7 ± 15.9	1.0	0.86
Diastlic Blood Pressure (mmHg)	74.8 ± 9.6	74.7 ± 9.8	0.9	0.87
HbA1c (%)	6.0 ± 0.5	6.0 ± 0.5	2.6	0.71
LDL (mg/dl)	118.0 ± 27.1	119.2 ± 28.8	4.5	0.40
HDL (mg/dl)	59.3 ± 14.7	59.2 ± 15.0	0.3	0.95
TG (mg/dl)	121.2 ± 66.8	123.2 ± 69.9	2.8	0.59
GOT (IU/L)	24.2 ± 8.2	24.6 ± 9.4	4.3	0.37
GPT (IU/L)	22.3 ± 11.5	23.3 ± 14.3	6.7	0.18
G-GTP (IU/L)	34.4 ± 36.1	35.9 ± 37.5	4.1	0.43
Uric acid (mg/dl)	5.5 ± 1.3	5.5 ± 1.3	0.8	0.88
eGFR (mL/min/1.73 m2)	71.6 ± 13.6	72.1 ± 14.9	2.9	0.57
Albmin (g/dl)	4.3 ± 0.3	4.3 ± 0.3	0.8	0.88
Hemoglobin (g/dl)	14.0 ± 1.3	14.0 ± 1.4	0.7	0.89

*SD* Standard deviation, *BMI* Body mass index, *HbA1c* Hemoglobin A1c, *LDL* Low-density lipoprotein, *HDL* High-density lipoprotein, *TG* Triacylglycerol, *AST* Aspartate aminotransferase, *ALT* Alanine aminotransferase, *GGT* γ-glutamyl transpeptidase, *eGFR* Estimated glomerular filtration rate, *CKD* Chronic kidney disease

**Fig. 2:**
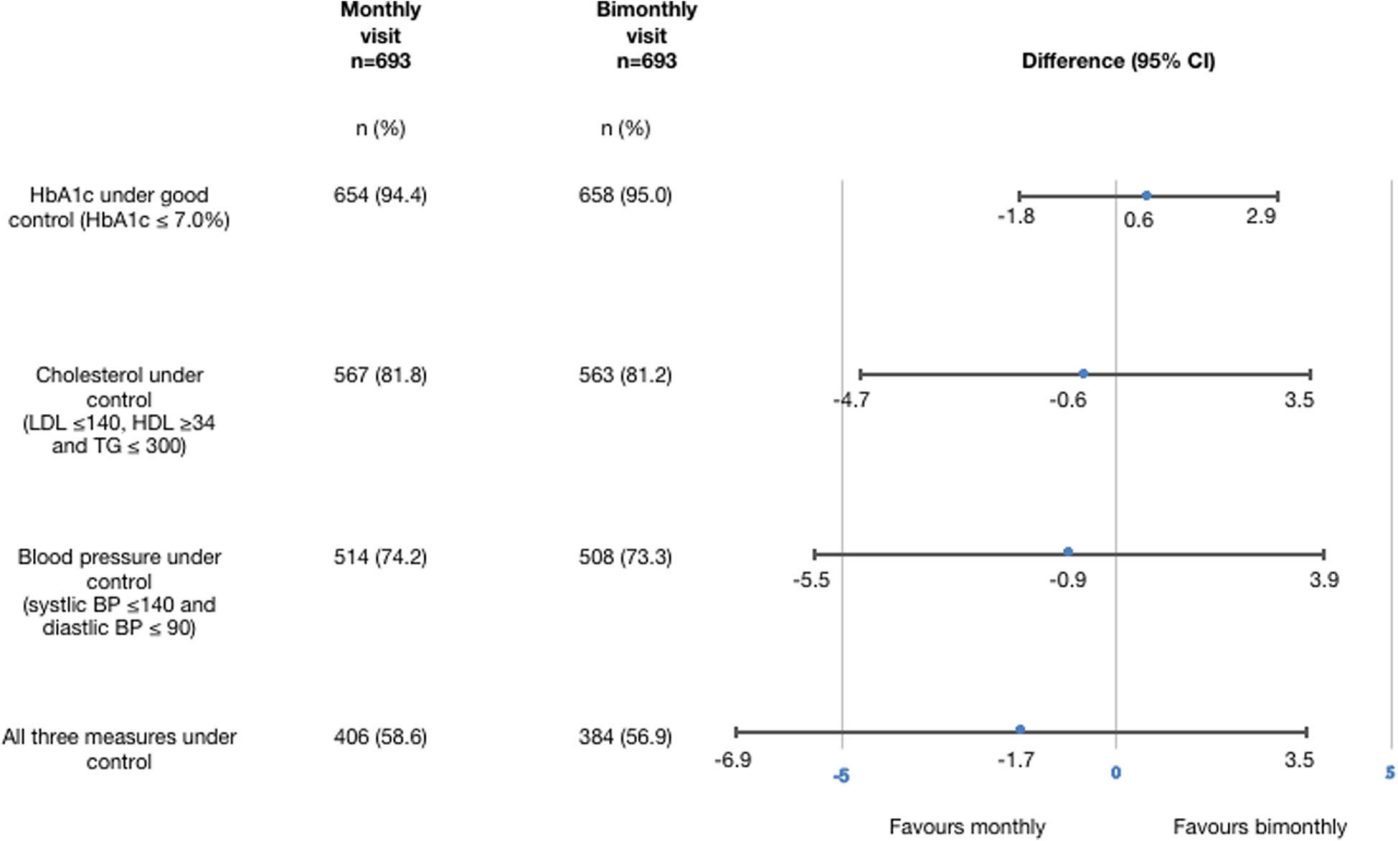
Assessment of equivalence. If the entire confidence interval is between the range − 5 and 5, monthly and bimonthly follow-up can be considered equivalent

In the monthly follow-up group, 654 (94.4%) remained under good diabetic control versus 658 (95.0%) in the bimonthly group. The CI was within the prespecified range (difference: 0.6%; 95% CI: − 1.8 to 2.9%), meaning that the result was equivalent (Fig. 2).

The proportion of patients with well-controlled hyperlipidemia was equivalent between monthly and bimonthly follow-up groups. The differences of the proportions of patients with blood pressure under well control and with all three outcomes (HbA1c, cholesterol and blood pressure) under well control between monthly and bimonthly follow-up were not within the range of equivalence, but there was not a significant difference between the two groups.

All other secondary health-related outcomes including HbA1c, systolic blood pressure, diastolic blood pressure, LDL cholesterol, HDL cholesterol, and triglyceride on a continuous scale, body mass index (BMI), the percentage of current smokers, the percentage of those with daily alcohol intake, and those who sleep well were equivalent except the percentage of persons doing regular exercise (Table 3). There was a significant difference between monthly and bimonthly follow-up regarding mean annual medical costs for diabetes care, which were 161,294 (standard deviation = 151,826) Japanese yen (JPY) and 96,292 (standard deviation = 179,272) JPY, respectively.

**Table 3:** Next-year results for the monthly and bimonthly follow-up groups

	Monthly follow-up (*n* = 693)		Bimonthly follow-up (n = 693)		
	Baseline, mean ± SD or n (%)	Next year, mean ± SD or n (%)	Baseline, mean ± SD or n (%)	Next year, mean ± SD or n (%)	Bionthly - monthly difference (95% CI)^a^
HbA1c (%)	6.0 ± 0.5	6.1 ± 0.6	6.0 ± 0.5	6.1 ± 0.5	−0.02 (−0.07 to 0.03)
Systolic blood pressure (mmHg)	129.6 ± 15.6	129.4 ± 14.7	129.7 ± 15.9	129.8 ± 15.3	0.26 (−1.32 to 1.83)
Diastolic blood pressure (mmHg)	74.8 ± 9.6	74.4 ± 9.4	74.7 ± 9.8	74.4 ± 9.4	0.11 (− 0.87 to 1.09)
LDL (mg/dl)	118.0 ± 27.1	115.9 ± 25.5	119.2 ± 28.8	116.5 ± 26.8	−0.61 (−3.06 to 1.83)
HDL (mg/dl)	59.3 ± 14.7	59.7 ± 15.2	59.2 ± 15.0	59.6 ± 15.3	0.01 (−0.79 to 0.81)
TG (mg/dl)	121.2 ± 66.8	125.9 ± 77.1	123.2 ± 69.9	124.3 ± 67.8	−3.6 (−10.28 to 3.08)
BMI	23.4 ± 3.0	23.4 ± 3.0	23.4 ± 3.2	23.4 ± 3.2	−0.06 (−0.13 to 0.02)
Current smoker (%)	63 (9.1)	65 (9.4)	71 (10.3)	70 (10.1)	−0.43 (−2.11 to 1.24)
Regular exercise (%)	334 (48.2)	356 (51.5)	348 (50.2)	365 (52.8)	−0.73 (−5.46 to 4.00)^b^
Sleep well (%)	595 (85.9)	588 (85.3)	598 (86.3)	590 (85.5)	−0.43 (−4.16 to 3.29)
Daily alcohol intake (%)	141 (20.4)	142 (20.5)	150 (21.7)	149 (21.5)	−0.29 (−2.45 to 1.87)
Annual cost (JPY)		161,294 ± 151,826		96,292 ± 179,272	− 6500.17 (− 8253.53 to − 4746.80)§

*SD* Standard deviation, *CI* Confidence interval, *HbA1c* Hemoglobin A1c, *BMI* Body mass index, *LDL* Low-density lipoprotein, *HDL* High-density lipoprotein, *TG* Triacylglycerol

^a^The differences in follow-up measurements were corrected for baseline measurement except annual cost

^b^Not within equivalence range

## Discussion

This study investigated the follow-up interval in well-controlled diabetes patients. HbA1c test results in the subsequent year were found to be equivalent in the monthly and bimonthly follow-up groups. Equivalence was also ascertained in other outcomes including BMI, systolic and diastolic blood pressure on continuous scale, and lifestyle-related status including smoking, regular exercise, sleep, and alcohol intake, as well as laboratory values including LDL cholesterol, HDL cholesterol, and triglycerides. Annual costs of care were significantly higher in the monthly follow-up group than in the bimonthly group.

These findings are compatible with the previous RCT in which 3-monthly and 6-monthly follow-up did not show a clinically relevant difference among patients with well-controlled diabetes mellitus [10]. In this RCT, patients were seen by nurse practitioners and only patients who did not have a preference for a follow-up interval were included, so the clinical situation differed from that of the present study. In both studies for patients already at the treatment goal shorter follow-up made no difference in maintaining good control. In the interim, other studies have demonstrated an association between shorter follow-up interval and better clinical outcomes. In these studies the participants were patients with uncontrolled diabetes [19] or diabetes patients regardless of diabetic control [9]; moreover, the outcome was improvement, not maintenance, of the HbA1c level. We assume that these differences in the study design led to different results.

The increasing cost of diabetes care has laid a heavy economic burden on society [20]. In 2014, the direct annual cost of diabetes was 825 billion USD globally, with Japan accounting for the fourth largest cost in the world at 37 billion USD [21]. Several studies have suggested that resources for diabetes care are overused and that patients visit health care facilities and undergo tests more often than needed, increasing both the treatment burden on patients and health care costs [15, 22, 23]. The cost of care is expected to be reduced by reducing the frequency of follow-up. Schectman et al. have shown that physicians can extend the follow-up interval without reducing patient satisfaction [24], implying that a longer follow-up interval would not be detrimental to diabetes control or patients’ health.

While guidelines across countries recommend that follow-up intervals for patients with type 2 diabetes be 3 months or longer, Japanese patients have a clinical consultation almost every month. Health care policy should thus be amended in order to change this practice. Japan’s health care system incentivizes doctors to conduct frequent consultations by providing a monthly lifestyle-associated disease management fee. Moreover, supplier-induced demand for diabetes care is indicated under the fee-for-service payment system [2]. Patients in turn tend to prefer frequent consultation because the universal health insurance allows them to pay only a small portion of the total consultation fee out of their pocket [5]. Doctors and patients thus have sufficient reasons to practice frequent consultation under the current health care system although such reasons do not derive from true clinical outcomes. Hence, the policy should be modified based on the evidence in order to reduce unnecessary expense and make diabetes care more cost-effective.

Several limitations of this study should be acknowledged. First, although we included variables that could determine the follow-up intervals and constructed a propensity score matched model, there may be other unmeasured factors that affect the follow-up intervals. For example, patients’ social aspects such as education status, economic status, and accessibility to health care facilities were not measured in the study. Matched pairs between monthly and bimonthly follow-up may not be well balanced after including these unmeasured variables. However, one study investigated whether patient demand-side factors explain regional variations in spending and found that patient demand-side was relatively minor in explaining variations [25]. Hence, these variables are not expected to considerably affect the follow-up interval. Added to this, our study do not include information on supplier side factors such as physician availability in the area, which have been shown to be associated with follow-up interval [2].

Second, the results of propensity score matching are generalizable only among those in the range of propensity scores included in the paired analysis, and may not be applicable to persons outside this range. For example, some patients from the monthly follow-up group with high BMI were excluded when they were matched to the bimonthly group, meaning that the results from this research might not apply to the obese population. In addition, we included only participants who had well-controlled diabetes mellitus and underwent annual health checkups over 2 consecutive years, differing somewhat from the general population.

## Conclusion

Patients with controlled diabetes mellitus who attend monthly or bimonthly follow-ups have equivalent HbA1c control. By reducing the frequency of follow-up, patients and physicians can reduce the social burden of diabetes mellitus.

## Abbreviations

ALT: Alanine aminotransferase
AST: Aspartate aminotransferase
CI: Confidence interval
eGFR: Estimated glomerular filtration rate
GGT: γ-glutamyl transpeptidase
HbA1c: Hemoglobin A1c
HDL: High-density lipoprotein
LDL: Low-density lipoprotein (LDL)
OECD: Organization for Economic Co-operation and Development (OECD)
RCT: Randomized controlled trial
